# Physiological costs of infection: herpesvirus replication is linked to blood oxidative stress in equids

**DOI:** 10.1038/s41598-018-28688-0

**Published:** 2018-07-09

**Authors:** David Costantini, Peter A. Seeber, Sanatana-Eirini Soilemetzidou, Walid Azab, Julia Bohner, Bayarbaatar Buuveibaatar, Gábor Á. Czirják, Marion L. East, Eva Maria Greunz, Petra Kaczensky, Benjamin Lamglait, Jörg Melzheimer, Kenneth Uiseb, Alix Ortega, Nikolaus Osterrieder, Ditte-Mari Sandgreen, Marie Simon, Chris Walzer, Alex D. Greenwood

**Affiliations:** 1 UMR 7221 CNRS/MNHN, Muséum National d’Histoire Naturelle, Sorbonne Universités, 7 rue Cuvier, 75005 Paris, France; 2 Institute for Biodiversity, Animal Health and Comparative Medicine, University of Glasgow, G12 8QQ Glasgow, Scotland UK; 3 Department of Biology, University of Antwerp, Universiteitsplein 1, 2610 Antwerp, Belgium; 4 Department of Wildlife Diseases, Leibniz Institute for Zoo and Wildlife Research, Alfred-Kowalke-Straße 17, 10315 Berlin, Germany; 5 Institut für Virologie, Robert von Ostertag-Haus, Zentrum für Infektionsmedizin, Freie Universität Berlin, Robert-von-Ostertag-Str. 7-13, 14163 Berlin, Germany; 6 Department of Reproduction Management, Leibniz Institute for Zoo and Wildlife Research, Alfred-Kowalke-Straße 17, 10315 Berlin, Germany; 7 Wildlife Conservation Society, Mongolia Program, Ulaanbaatar, Mongolia; 8 Department of Ecological Dynamics, Leibniz Institute for Zoo and Wildlife Research, Alfred-Kowalke-Straße 17, 10315 Berlin, Germany; 9 Center for Zoo and Wild Animal Health, Copenhagen Zoo, Roskildevej 38, 2000 Frederiksberg, Denmark; 10 Parc Zoologique de Thoiry, Rue du Pavillon de Montreuil, 78770 Thoiry, France; 11 Research Institute of Wildlife Ecology, University of Veterinary Medicine, Savoyenstrasse 1, A-1160 Vienna, Austria; 12 Norwegian Institute for Nature Research – NINA, Sluppen, NO-7485, Trondheim, Norway; 13 Faculty of Veterinary Medicine, Université de Montréal, 3200 Rue Sicotte, Saint-Hyacinthe, Québec, J2S 2N4 Canada; 14 Réserve Africaine de Sigean, 19 Chemin Hameau du Lac, RD 6009, 11130 Sigean, France; 15 Department of Evolutionary Ecology, Leibniz Institute for Zoo and Wildlife Research, Alfred-Kowalke-Straße 17, 10315 Berlin, Germany; 16 Ministry of Environment and Tourism, Private Bag 13301, Windhoek, Namibia; 17 Givskud Zoo, Løveparkvej 3, Givskud, 7323 Give, Denmark; 18 Wildlife Conservation Society, 2300 Southern Blvd., 10460 Bronx, New York USA; 19 Department of Veterinary Medicine, Freie Universität Berlin, Oertzenweg 19, Berlin, 14163 Germany

## Abstract

Viruses may have a dramatic impact on the health of their animal hosts. The patho-physiological mechanisms underlying viral infections in animals are, however, not well understood. It is increasingly recognized that oxidative stress may be a major physiological cost of viral infections. Here we compare three blood-based markers of oxidative status in herpes positive and negative individuals of the domestic horse (*Equus ferus caballus*) and of both captive and free-ranging Mongolian khulan (*Equus hemionus hemionus*) and plains zebra (*Equus quagga*). Herpes positive free-ranging animals had significantly more protein oxidative damage and lower glutathione peroxidase (antioxidant enzyme) than negative ones, providing correlative support for a link between oxidative stress and herpesvirus infection in free-living equids. Conversely, we found weak evidence for oxidative stress in herpes positive captive animals. Hence our work indicates that environment (captive versus free living) might affect the physiological response of equids to herpesvirus infection. The Mongolian khulan and the plains zebra are currently classified as near threatened by the International Union for Conservation of Nature. Thus, understanding health impacts of pathogens on these species is critical to maintaining viable captive and wild populations.

## Introduction

Pathogens pose a serious threat to domestic animal and wildlife health^1–3^. Infections may have a number of negative effects such as a reduced capacity to forage, avoid predators, reproduce and maintain homeostasis^1,4,5^. For example, viral infections may dramatically increase the mortality rate in mammalian populations (e.g., border disease virus^6^; canine distemper virus^7^).

Herpesviruses are a large family of DNA viruses widespread across environments and animal species. Herpesviruses are extremely efficient at establishing chronic infections^8^ and may cause severe disease in both domestic and free-ranging animals^9,10^. After initial lytic replication, the hallmark of herpesvirus infection is the establishment of latency during which genome maintenance is ensured but productive replication is suppressed. Exposure of hosts to environmental stressors or hormonal changes may result in reactivation and virus shedding^9,10^. Viral replication is generally only recognized when clinical manifestations appear, but clinical signs might not be obvious if the organism rapidly mounts an immune response and controls virus replication. Moreover, sick individuals might be killed by predators well before the clinical signs are manifested.

Irrespective of the appearance of clinical signs, the replication of herpesviruses may have less obvious costs for the organism in terms of tissue damage, reduced reproduction or survival. Experimental and meta-analytical evidence from domestic animals and humans suggested that oxidative stress might be an important physiological cost of herpesvirus infection^11–15^. Herpesvirus infection may be associated with an increased production of reactive oxygen species, molecular oxidative damage and changes in both non-enzymatic and enzymatic antioxidant defences^11–15^. Herpesvirus infected cell cultures show an accumulation of protein carbonyls (i.e., protein oxidative damage) during the progress of infection, which is important since only a small fraction of carbonylated proteins are removed through proteasome-dependent proteolysis^16^. Protein carbonylation occurs when carbonyls (C=O) are introduced into proteins through the reactions with free radicals or lipid peroxidation products (malondialdehyde and hydroxynonenal). Carbonylation is mostly irreversible and results in alteration of protein structure and function^17^. Experimental infection of cell lines has also shown that the activities of antioxidant enzymes may decrease over time^18,19^ and that chemical stimulation from reactive oxygen species may even induce an upregulation of antioxidant enzymes in order to counteract oxidative stress^17^. Mounting an antioxidant response may entail costs (e.g., energetic, nutrients for enzyme synthesis, production of enzymatic co-factors), which implies that factors such as the environmental conditions animals live in might play an important role in mediating an organism’s response to the infection.

Meta-analytical evidence has further demonstrated that oxidative stress is consistently increased by herpesvirus infection across multiple tissues and host species infected with different herpesviruses^15^. Moreover, meta-analytical evidence also showed that the administration of antioxidants to animals with a clinical infection reduced herpesvirus yield, indicating that oxidative stress might be favorable for virus replication^15^.

Evidence that oxidative stress is a physiological cost associated with herpesvirus infection in wildlife is lacking. Recent work on magnificent frigatebird (*Fregata magnificens*) nestlings demonstrated that individuals with visible clinical signs of disease and high herpesvirus replication had higher levels of oxidative stress than individuals without clinical signs^20^. These findings suggested that there is an uncharacterized link between oxidative stress and herpesvirus infection in wildlife. This missing link about the roles of molecular oxidative damage caused by pro-oxidant chemicals, and the antioxidant mechanisms as mediators of the costs of immune activation induced by anti- and pro-inflammatory mechanisms is an important gap in current knowledge^21,22^. A key hypothesis of immuno-oxidative ecology is that oxidative stress provides a means to quantify the physiological costs of infections and/or immune activity because there is increasing evidence that oxidative stress does affect key homeostatic mechanisms and life-history traits^22^.

Equidae are particularly suitable to test the extent to which herpesvirus infection is associated with oxidative stress and whether such an association is stronger in free-ranging than captive individuals. Equidae host many different herpesviruses from the two subfamilies *Alphaherpesvirinae* and *Gammaherpesvirinae*. Alphaherpesviruses (EHV-1, EHV-4 and EHV-9) can cause respiratory disease, abortion, neonatal death and myeloencephalopathy with significant economic consequences worldwide^23^. EHV-1 is arguably one of the most important equine pathogens with a worldwide distribution in domestic horses (*Equus ferus caballus*), but it or closely related herpesviruses can also occur in other equids including several species of zebra, domestic donkeys and onagers^24–27^. EHV-9 is similarly important and widespread as EHV-1, but it is apparently absent from domestic horses^27^. Although the clinical importance of gammaherpesviruses (e.g., EHV-2 or EHV-5) is less clearly defined, several reports found that they may be associated with respiratory disease^28,29^ or multinodular pulmonary fibrosis^30^.

In this study, we have measured the presence of replicating herpesviruses and three blood-based markers of oxidative status in domestic horses, in both captive and free-ranging Mongolian khulan (*Equus hemionus hemionus*) and plains zebras (*Equus quagga*) to assess whether: (i) herpesvirus positive individuals have higher oxidative protein damage than individuals that do not harbour herpesviruses (i.e., herpes negative); (ii) herpesvirus positive individuals have lower activity of two antioxidant enzymes (GPX and SOD) than negative individuals when costs of antioxidant upregulation are high; (ii) the degree to which herpesvirus infection causes oxidative stress varies among herpesviruses (strain-related stress hypothesis); and (iii) life history theory predicts that food/antioxidant intake will determine resource allocation to immune and stress responses, thus effect size differences in oxidative damage and antioxidant protection should be smaller between herpes positive and negative captive individuals than con-specific free-living individuals because captive animals have a better supply of energy and nutrients.

## Results

### Captive animals

Thirteen of 28 horses were PCR positive for one or more of the following herpesviruses, EHV-1, EHV-2 and EHV-5 (Table 1). Nine horses were co-infected by two or three herpesvirus strains (Table 1). Eight out of 18 captive khulan were positive for herpesvirus Wild Ass Gamma HV or EHV-9. Sixteen out of 18 captive zebras were positive for herpesvirus EHV-5, EHV-7, EHV-9 or Zebra Gamma HV (Table 1). None of the captive zebras were infected with more than one herpesvirus strain.

**Table 1:** Number of positive animals (n) for each herpesvirus strain identified. The number of plains zebras from Namibia having a given strain is reported between brackets with the letter N (e.g., 1N means 1 plains zebra from Namibia).

Species	Herpes strain(s)	n
Horse	EHV-1	1
	EHV-2	2
	EHV-5	1
	EHV-1 + EHV-2	1
	EHV-1 + EHV-5	2
	EHV-2 + EHV-5	4
	EHV-1 + EHV-2 + EHV-5	2
Captive khulan	Wild ass Gamma HV	6
	EHV-9	2
Captive zebra	EHV-5	1
	EHV-7	10
	EHV-9	4
	Zebra Gamma HV (=Equus zebra HV)	1
Free-ranging khulan	AHV 5	4
	Wild ass Gamma HV	4
	EHV-1	1
	EHV-7	1
	EHV-9	1
	AHV-5 + EHV-7	2
	Wild ass Gamma HV + EHV-7	2
Free-ranging zebra	AHV-5	8^(1N)^
	EHV-2	1
	EHV-5	3^(3N)^
	EHV-7	4
	EHV-9	2^(1N)^

**Figure 1:**
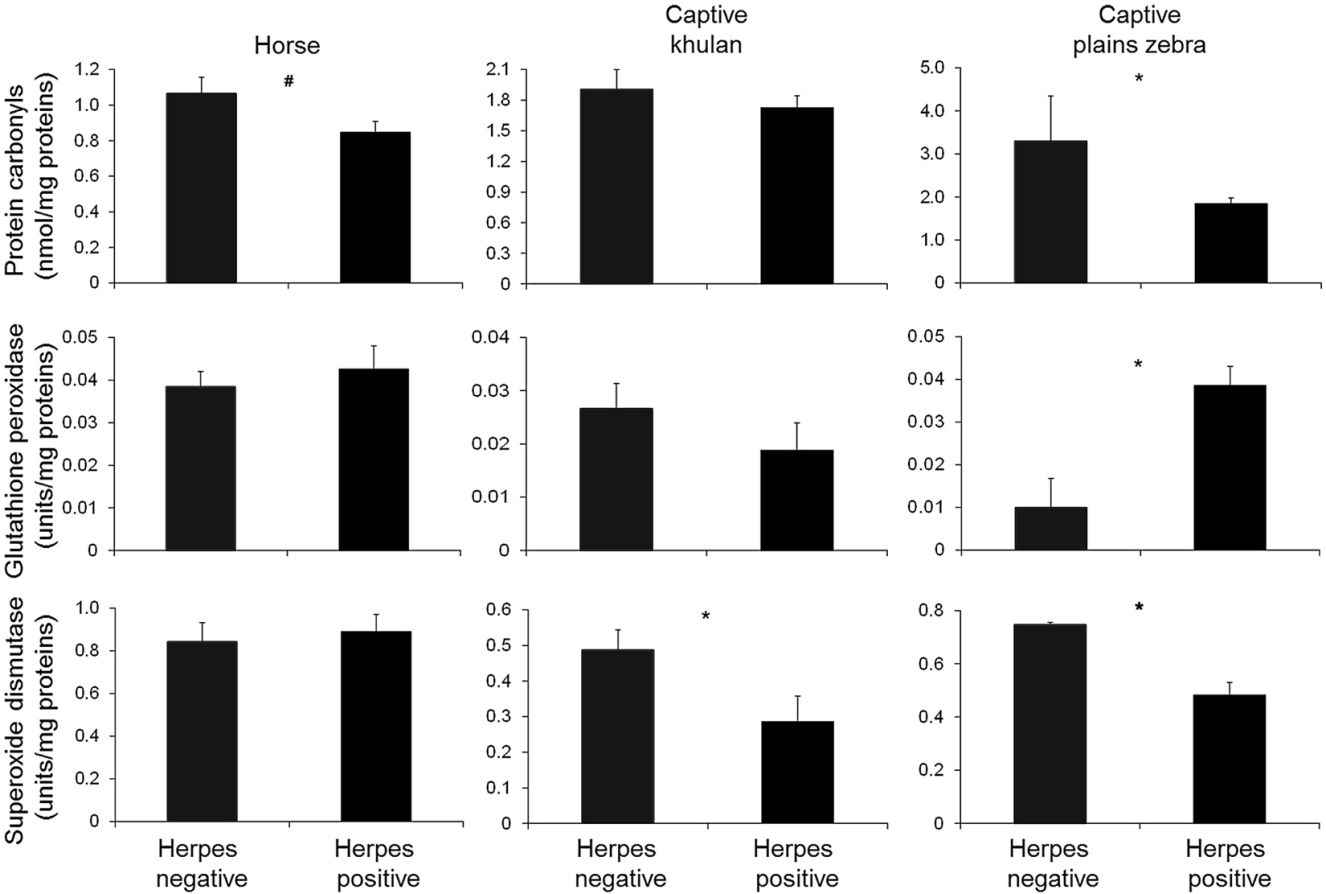
Histograms showing means and standard errors of oxidative status markers measured in captive animals. Note that for captive plains zebras we reported means rather than medians to enable a direct comparison of marker values with the other groups and literature. The symbol * indicates a *P*-value ≤ 0.05; the symbol # = 0.057.

There was a tendency for positive horses to have lower (square-root transformed) protein carbonyls than negative horses (*t*-test = −1.99, *P* = 0.057; Fig. 1). The activities of (square-root transformed) GPX and SOD were similar between positive and negative horses (GPX, *t*-test = 0.55, *P* = 0.59; SOD, *t*-test = 0.39, *P* = 0.70; Fig. 1). Markers did not differ between horses co-infected by two or three strains and those infected by a single strain (*t*-test, *P* ≥ 0.22). Horses infected with an alphaherpesvirus strain had marginally higher (square-root transformed) protein carbonyls than those infected with gammaherpesvirus strains (*t*-test = −2.20, *P* = 0.050), while GPX and SOD activities did not differ between them (*t*-test, *P* ≥ 0.40).

Herpes positive captive khulan had lower SOD values than negative captive khulan (*t*-test = 2.25, *P* = 0.039; Fig. 1), while positive and negative captive khulan had similar protein carbonyls and GPX (protein carbonyls, *t*-test = 0.76, *P* = 0.46; GPX, *t*-test = 1.13, *P* = 0.28; Fig. 1).

Herpes positive captive zebras had lower protein carbonyls (Mann-Whitney, *Z* = 1.90, *P* = 0.052; Fig. 1), higher GPX (Mann-Whitney, *Z* = −2.04, *P* = 0.026; Fig. 1) and lower SOD (Mann-Whitney, *Z* = 2.18, *P* = 0.013; Fig. 1) than negative captive zebras. Values of GPX activity of the two negative zebras were above the 75 percentile, while values of both protein carbonyls and SOD activity were below the 25 percentile. None of the oxidative stress status markers differed between zebras infected with alpha or gammaherpesvirus strains (*P* ≥ 0.14).

### Free-ranging animals

Fourteen of 21 free-ranging khulan were positive for one or more of the following herpesviruses AHV-5, Wild Ass Gamma HV, EHV-1, EHV-7 and EHV-9 (Table 1). Three khulan were co-infected by two herpesvirus strains (Table 1). Eighteen of 27 free-ranging plain zebras were positive for one or more of the following herpesviruses AHV-5, EHV-2, EHV-5, EHV-7 and EHV-9 (Table 1). No zebra was infected by more than one herpesvirus strain.

**Figure 2:**
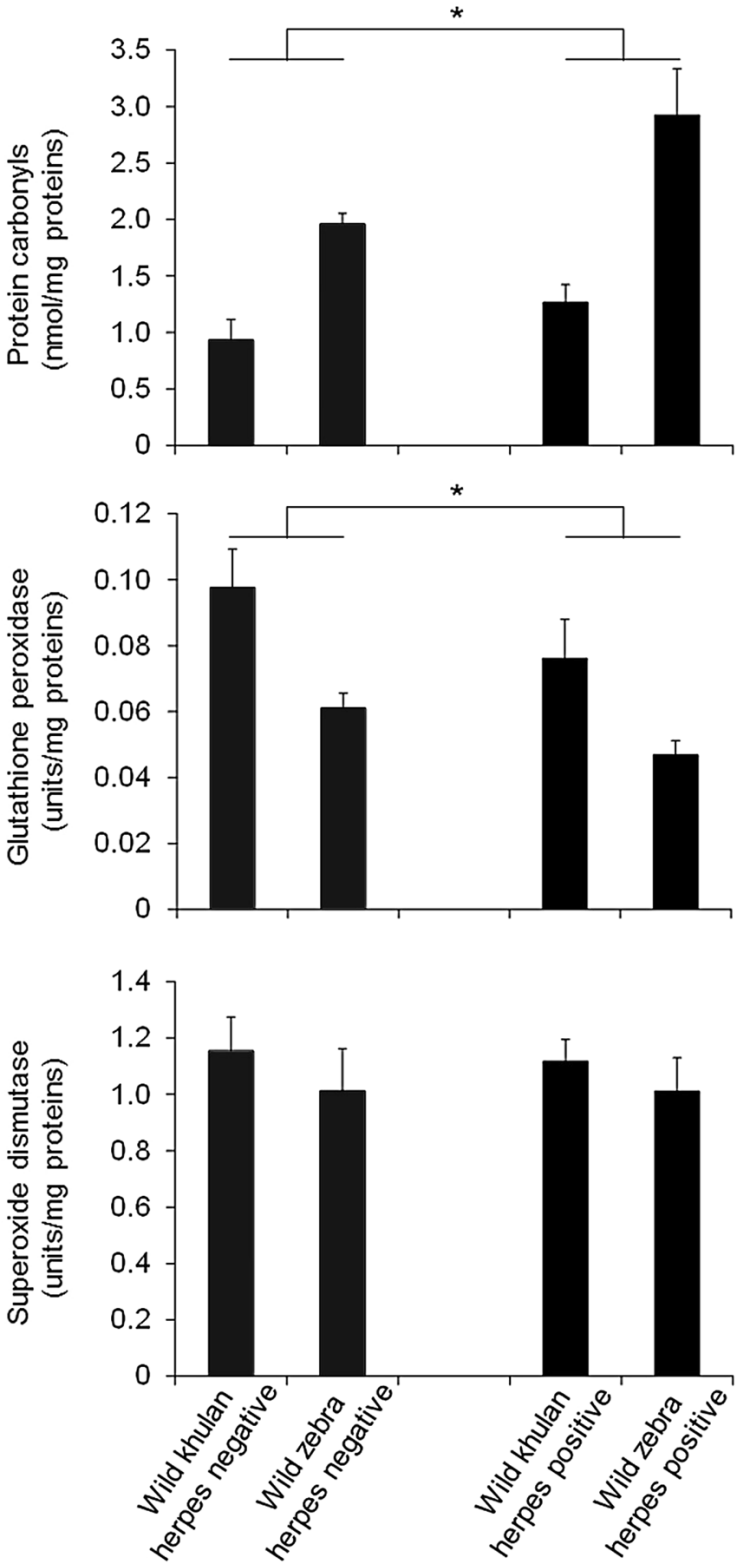
Histograms showing least square means and standard errors of oxidative status markers measured in free-living animals. *Indicates a *P*-value < 0.05. Note that the p-value refers to the comparison between positive and negative animals irrespective of the species according to model’s outcomes.

Free-ranging zebras and khulan had similar patterns of variation in oxidative status, with positive individuals having higher protein carbonyl values (GLZ, coefficient estimate, 0.32 ± 0.13SE, *P* = 0.016; Table 2, Fig. 2) and lower GPX (GLZ, coefficient estimate, 0.009 ± 0.004SE, *P* = 0.041 Table 2, Fig. 2) than negative individuals. The activity of SOD was similar between positive and negative individuals irrespective of the species (Table 2, Fig. 2).

**Table 2:** Outcomes of generalized linear models including free-ranging khulan and zebras.

Variable	Factor	Wald	P
Protein carbonyls	Species	24.87	**<0.001**
	Herpes	5.82	**0.016**
	Species × Herpes	1.39	0.239
Glutathione peroxidase	Species	14.22	**<0.001**
	Herpes	4.18	**0.041**
	Species × Herpes	0.18	0.674
Superoxide dismutase	Species	1.02	0.313
	Herpes	0.03	0.873
	Species × Herpes	0.02	0.889

Herpes refers to positive and negative animals. Significant *P*-values are shown in bold.

### Estimates of effect sizes

**Figure 3:**
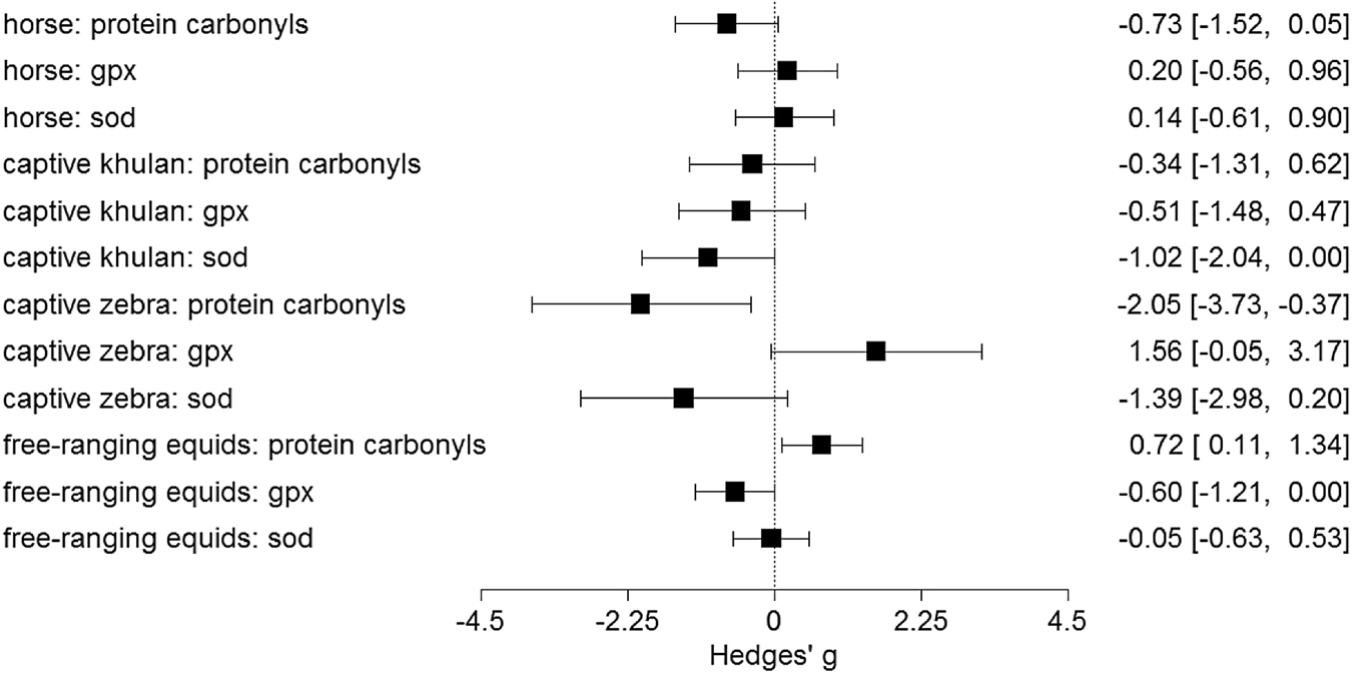
Estimates of effect size (standardized measure of the magnitude of a relationship) and 95% confidence interval calculated from all test statistics. Estimates are positive when values of a given marker are higher in herpes positive than negative animals.

Most estimates of effect sizes were intermediate to large. Only those of (i) protein carbonyls in both captive zebras and free-ranging equids did not overlap zero and (ii) SOD in captive khulan and GPX in free-ranging equids included zero in the confidence interval (Fig. 3).

## Discussion

The link between blood oxidative status and herpesvirus infection was contingent on the environmental conditions, captive versus free living. Herpes positive free-ranging animals had significantly more protein oxidative damage (protein carbonyls) and reduced GPX than negative free-ranging animals. In contrast, only a weak association was found between oxidative stress and herpes infection in captive animals. Effect size estimates (Fig. 2) were intermediate to large, thus explaining 9 to 25% of the variance^31^. Intermediate effect sizes are considered biologically meaningful because average proportions of variance explained in physiological, ecological and evolutionary studies are usually below 7%^32^. This is not surprising given that free-living animals are affected by numerous factors, thus the amount of variance explained by any single factor is expected to be low. Our interpretation of the biological relevance of our effect size estimates is further supported by several laboratory studies showing that when protein carbonyls accumulate, they tend to aggregate and can lead to cell death, tissue injury and development of disorders^33,34^. Thus, our data might suggest that prolonged or recurrent activity of herpesvirus might negatively impact fitness-related traits (e.g., fertility, cellular ageing) in free-ranging equids.

In contrast positive captive equids had protein carbonyls similar to negative ones or even lower. This result might indicate that herpes activity did not alter the blood oxidative balance or that animals have upregulated proteolysis of protein carbonyls to avoid their accumulation. It is unclear why the SOD activity was lower in positive captive khulan than in negative ones because the amount of damage was similar between them. In zebras, both the amount of damage and the SOD activity were actually lower in positive captive animals than in negative ones. SOD protects cells from the pro-oxidant action of the superoxide free radical^17^, thus a reduced SOD protection may translate in increased protein oxidative damage. It might be that the lower SOD activity of positive animals reflects excessive consumption of the enzyme (antioxidant consumption hypothesis) to buffer increased superoxide production rather than an inhibitory effect (antioxidant inhibition hypothesis) of viral infection on the enzyme^35^. Consistent with the antioxidant consumption hypothesis, previous work on cattle (*Bos taurus domesticus*) found that individuals naturally infected with bovine herpesvirus type 1 (*Alphaherpesvirinae*) showed mild to severe respiratory signs and lower serum non-enzymatic antioxidants than herpes negative animals. However, they had serum oxidative damage similar to negative animals^36^. Another factor might be the activity of GPX. Although it was similar between positive and negative captive khulan, it was significantly higher in positive captive zebras compared to herpes negative ones. The upregulation of GPX might explain why in zebras, but not in khulan, the amount of damage was lower in positive than negative animals. GPX actually eliminates lipid hydroperoxides from cells, which are precursors of several compounds (e.g., malondialdehyde) that may cause protein carbonylation^17^. Costs associated with GPX upregulation might further explain differences between captive khulan and zebras or free-ranging equids. Further work is needed to clarify the exact mechanisms modulating the different antioxidant responses of the two species during herpesvirus infection, the costs associated with the antioxidant response, and which factors (e.g., species, environment, life-history stage) affect the magnitude of such costs.

Differences in herpesvirus strain and their frequency between captive and free-ranging animals may explain the different oxidative status between them. Differences in virulence and pathological consequences form an important basis for distinguishing between different herpesvirus strains. For example, compared to the *Gammaherpesvirinae*, herpesvirus strains belonging to the *Alphaherpesvirinae* (EHV-1, EHV-4 and EHV-9) have well documented detrimental effects on the host, being responsible for severe respiratory syndromes in equids^23^. However, our data did not support the strain-related stress hypothesis because *Alphaherpesvirinae* were more common in captive than free-living individuals. *Alphaherpesvirinae* occurred in 46.2% of horses and 25% of both captive khulan and plains zebras, while they occurred only in 14.3% and 11.1% of free-ranging khulan and plains zebras, respectively. Our results on the weak connection between herpesvirus strain and oxidative stress are in agreement with a previous meta-analysis, which found that the effect size of herpesvirus infection on oxidative stress markers was similar across different herpesvirus strains^15^. Further work is needed to clarify whether strains differ in how they impact oxidative status. For example, we do not know whether (i) such an effect is localized to the tissue where the strain is more active or (ii) the rate at which oxidative status markers change differs among strains, depending on their replication rate.

The stronger link between oxidative status and herpesvirus infection in free-ranging than captive equids might be explained by the constraints of the environment the animals lived in. Resources (e.g., nutrients, energy, antioxidants) are limited, thus allocation of a key resource to one trait, such as resistance against viral infection, means that less can be allocated to other functions that might be important in terms of Darwinian fitness (e.g., fertility). Captive animals were maintained under standard husbandry conditions (e.g., *ad libitum* food, absence of predators and competitors), which relax the constraints imposed by tradeoffs among competing functions. In contrast, resources are generally more limited for free-ranging animals imposing greater constraints. Moreover, free-ranging animals might also have been exposed to concomitant stressors that would strengthen the detrimental effects of a viral infection. For example, a meta-analysis of the effects of immune responses on oxidative status markers found that the effect size was always high in free-living birds, and more variable in captive birds^37^. This result may be explained by either the relative quality and quantity of food available or the fact that free-living birds cannot readily recover from the costs of an immune response because of the tradeoff between foraging and other energy demanding behaviors, such as mating and egg laying.

We could not assess whether herpesvirus infection associated oxidative stress differed between males and females. Prior work did not detect any significant sexual differences in oxidative status markers between herpesvirus-infected males and females in Magnificent frigatebird nestlings^20^. Sexual differences in oxidative status markers are also generally small to moderate in mammals, even if among species variation occurs^38^. We suggest that future studies should look at sex effects in more detail, comparing males and females across different demanding phases of life, where sexes might be faced with tradeoffs of greater or lesser magnitude.

## Conclusions

We demonstrated that blood oxidative stress was higher in herpes positive free-living equids than in herpes negative ones. In contrast, the link between oxidative stress and herpesvirus infection was much weaker in captive animals. Our results also suggest that neither herpesvirus strain nor co-infection seem important determinants of oxidative stress levels in blood. However, free-living animals harbored a larger number of strains than captive ones and the potential patho-physiological consequences for infected animals needs further consideration. It will also important to assess whether the link between herpesvirus infection and oxidative status is affected by the presence of co-infections with other viruses.

Integration of life-history theory with physiological and virological research is likely to advance our understanding of mechanisms that underlie disease state and the potential for long-term population level effects. Such an understanding might be important for effective programs of population health monitoring and management, a conservation priority for species like the Mongolian khulan and the plains zebra currently classified as near threatened by the International Union for Conservation of Nature.

## Materials and Methods

### Ethics statement

Collection of horse blood samples in Egypt was done according to the Ministry of Health and Population (General Administration of Medical Licenses; approval number 36490). Horse blood samples were imported from Egypt to Germany based on the import permit issued by Senatsverwaltung für Justiz und Verbraucherschutz (permission number: V A VET 0.2). The work on free-living khulan was approved by the ethical committee of the University of Veterinary Science in Vienna (ETK-15/03/2016) and the Mongolian Government (05/5656). Permission to conduct research in Namibia was granted to PAS by the Ministry of Environment and Tourism (permit No. 2094/2016). Permission to export sample material from Namibia was granted by an MET export permit (No. 105336), and samples were transported to Germany in compliance with the Nagoya Protocol on Access to Genetic Resources. Permission to conduct research in Tanzania was granted to PAS and MLE by the Tanzania Commission for Science and Technology (permit No. 2015-168-NA-90-130), and the Tanzanian Wildlife Research Institute. All experiments were performed in accordance with relevant guidelines and regulations.

### Sampling of captive animals

Whole blood samples of 28 (24 females and 4 males) clinically healthy Arabian horses were collected in Egypt (27 in Giza and 1 in Alexandria) in 2015. The Arabian horses were used mainly for show purposes. After collection, all blood samples were transported immediately to the laboratory keeping tubes at 4 °C while on route. Samples were initially stored at −20 °C in Egypt and subsequently at −80 °C at the Institut für Virologie, Freie Universität Berlin in Germany.

Samples of 21 captive khulan (17 females and 4 males) were taken in the Serengetipark Hodenhagen (Germany) in 2017. Whole blood samples were withdrawn from the jugular vein into EDTA tubes (Sarstedt, Nürnbrecht, Germany) 15–20 minutes following darting and stored at −20 °C in the Serengetipark. Thereafter the samples were transported to the IZW and stored at −80 °C until further evaluation. In addition, 2 UTM^TM^ Viral Transport Media swabs (Copan Diagnostics Inc., Murrieta, California, USA) were taken from the right and left nostril to test for acute nasal shedding of EHV.

Samples of 18 captive plains zebras were collected from the Réserve Africaine de Sigean (5 females and 4 males), Thoiry zoo (4 males) and Givskud zoo (2 females and 3 males), respectively. Blood sampling and storage were similar to those used for the captive khulans.

### Sampling of free-living animals

Blood samples of 21 (13 females and 8 males) adult free-living Mongolian khulan were collected in Southern Gobi (Mongolia) in October 2015. Blood samples from 27 adult free-ranging female plains zebras were collected in 2015 in the Etosha National Park (Namibia; n = 7) and in 2016 in the Serengeti National Park (Tanzania; n = 20). All khulan and plains zebras were in good physical condition and showed no visible clinical symptoms of EHV infection. We did not see any signs of nasal, ocular or similar discharge; any signs of diarrhea or infection with ecto-parasites; and any deep open wounds or broken bones. Both electrocardiogram and body temperature measured during the anesthesia were normal. The animals were immobilized with a dart gun^39^ and a sample of blood was drawn from the jugular vein into EDTA-tubes (S-Monovette K3E, Sarstedt, Hildesheim, Germany) approximately 15–30 minutes after darting. In order to screen for acute nasal shedding of EHV, two UTM^TM^ Viral Transport Media swabs (Copan Diagnostics Inc., Murrieta, California, USA) were rubbed firmly on the nasal mucosa of each animal.

Khulan samples were stored at −20 °C while on the field and were then transported to the Research Institute of Wildlife Ecology, Austria, in full compliance with the Convention on International Trade in Endangered Species (CITES), where samples were stored at −80 °C until transported to the IZW in Germany, where they were likewise stored until laboratory analyses. Zebra samples from Namibia and Tanzania were transported to the respective field laboratory in a cooling box at 4 °C and then stored and transported at −20 °C to the laboratory facilities of the IZW in Germany, where blood samples were stored at −80 °C.

### Herpesvirus screening

Viral DNA was extracted (Stratec Biomedical, Germany) from collected horse samples using a commercially available kit (QIAamp® DNA Blood Mini Kit, Giagen) as described previously^40–43^. All DNA samples were analyzed by qPCR with the Applied Biosystems 7500 FAST (ABI, Foster City, CA) using specific primers and probes targeting the gB gene of several herpesviruses^44,45^. Positive (virus-infected cell culture) and negative (water) controls were included from the beginning of the extraction procedure until the reading of the results. EHV-specific primers that target various regions of two genes, gB (ORF33), and/or POL (DNA polymerase; ORF30), were also employed. The amplified products were purified and directly sequenced by Sanger sequencing (LGC Genomics). Viral DNA was extracted from blood samples of both captive and free-ranging khulan using a commercially available kit (QIAamp® DNA Blood Mini Kit, Giagen) and from nasal swabs using the Invisorb® Spin Virus DNA Mini Kit, Stratec, according to the manufactures instructions. From samples of wild and captive zebras, viral DNA was extracted from blood samples and nasal swabs using a commercially available kit (NucleoSpin® Tissue, Macherey-Nagel, Düren, Germany) according to the manufacturer’s instructions for the respective sample type (blood or buccal swab protocol, respectively). To test for EHV DNA, we performed a nested PCR as described by^46^ with a modified thermocycling protocol^47^. Amplified products were visualized on a 1.5% agarose gel and purified using a kit (NucleoSpin® Gel and PCR Clean-up, Macherey-Nagel, Düren, Germany) according to manufacturer’s instructions, for subsequent Sanger sequencing. Purified PCR products were sequenced by LGC Genomics GmbH and virus strains identified by blast searches against GenBank entries. Animals were considered positive when at least one of the samples tested positive for herpesviruses. Analyses of horse samples were carried out at the Freie Universität Berlin. Analyses of khulan and plains zebra samples were carried out at the IZW in Berlin.

### Laboratory assays of oxidative status markers

One oxidative damage (protein carbonyls) and two antioxidant (GPX and SOD) blood-based markers were assessed in whole blood samples using commercially available kits commonly applied to mammals^48,49^ following the manufacturer’s instructions unless otherwise mentioned. These three markers were chosen because they are responsive to herpesvirus infection^15^. Protein carbonyls (marker of oxidative protein damage) were measured using the Protein Carbonyl Colorimetric assay (Cayman Chemical Company, Ann Arbor, MI, USA). Values were expressed as nmol per mg of protein. The Ransel assay (RANDOX Laboratories, Crumlin, UK) was used to quantify the activity of glutathione peroxidase (GPX), which is an antioxidant enzyme that detoxifies cells from peroxides and organic hydroperoxides. The activity of GPX was expressed as units of GPX per mg of protein. The Ransod assay (RANDOX Laboratories, Crumlin, UK) was used to quantify the activity of superoxide dismutase (SOD), which is an antioxidant enzyme that protects cells against the pro-oxidant action of superoxide free radicals. The activity of SOD was expressed as units of SOD per mg of protein. Quality controls with reference values of markers were used in all assays. The Bradford protein assay (Bio-Rad Laboratories, Hercules, USA), with albumin as a reference standard, was used to quantify the total concentration of protein in samples. All the analyses were carried out at the IZW in Berlin.

### Statistical analyses

The sample was heterogeneous in terms of species, origin of animals and sample size. Thus, we performed several subset analyses. Unpaired t-tests were used to compare herpes positive and negative horses and captive khulan, separately. Transformations of dependent variables were applied to achieve a normal distribution when needed. As for the captive zebras, the non-parametric Mann–Whitney U test was used because of the low and unbalanced sample size. Samples of herpes positive zebras were pooled together because all oxidative status markers were similar among zebras coming from the three zoos (*P* ≥ 0.21). Generalized linear models (GLZ) were used for data collected from free-ranging animals to assess relationships between each oxidative status marker and the predictor variables herpes infection (positive and negative) and species (khulan and zebra), as well as their interaction. Samples of plains zebras from Namibia and Tanzania were pooled because values in oxidative status markers between herpes positive and negative were similar in the two sampling areas for all the markers measured (sampling area × herpes, *P* ≥ 0.29). A gamma error function and an identity-link function were applied to models of protein carbonyls. A normal error function and an identity-link function were applied to models of GPX and SOD. Functions were selected on how well they fitted with the data using the Akaike Information Criterion. All analyses were performed using STATISTICA 10 (StatSoft, Tulsa, OK, USA). Finally, the compute.es package^50^ in R^51^ was used to calculate the standardized effect size Hedges’ g from test statistics. The forestplots function of the metafor package in R was used to visualise boxplots of effect size and 95% confidence interval. Effect sizes were considered to be small (Hedges g = 0.2, explaining 1% of the variance), intermediate (g = 0.5, explaining 9% of the variance) or large (g = 0.8, explaining 25% of the variance) according to^31^.

### Data availability

The datasets generated during and/or analysed during the current study are available from the corresponding author on reasonable request.
